# Ultra-Large Von Willebrand Factor and Platelets: Missing Links to the Pathogenesis of Aortic Valve Stenosis?∗

**DOI:** 10.1016/j.jacbts.2022.04.003

**Published:** 2022-07-25

**Authors:** Dong Chen

**Affiliations:** Division of Hematopathology, Mayo Clinic, Rochester, Minnesota, USA

**Keywords:** aortic stenosis, echocardiography, von Willebrand factor

**Figure:**
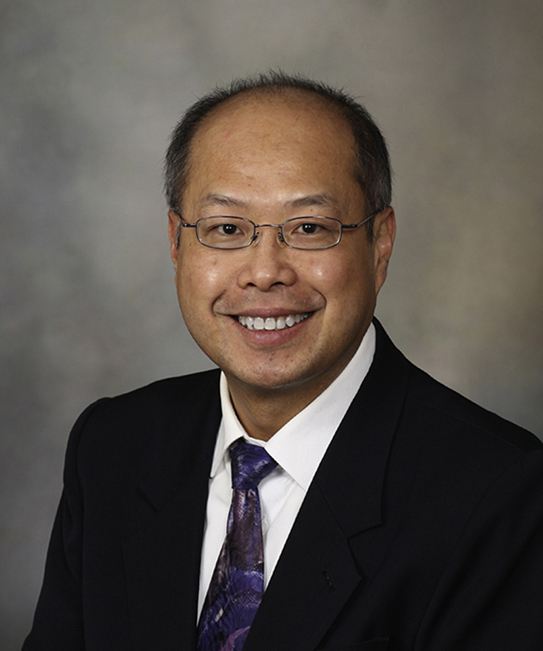

Roughly 1.5 million people in the United States suffer from aortic valve stenosis (AVS), whereas an estimated 250,000 patients with severe AVS are symptomatic and require clinical intervention. The fact that both prevalence and severity of AVS increase with age (>65 years) implies an underlying mechanism of age-related disease progression.^1^ The natural history of AVS is characterized by an initial slow course in patients with asymptomatic AVS and followed by a quick severity jump once symptoms develop. The initially postulated mechanism of AVS progression was passive wear and tear. However, recent studies have gradually pointed to an active pathophysiological process, which entails valve inflammation and interstitial cell and matrix proliferation. This mechanism shares some similarities with atherosclerotic vascular disease (AVD).^2^

Recent studies by Wu et al^2^ have shed some light on the potential roles of von Willebrand factor (vWF) on the pathophysiology of AVD. vWF is a large multimeric plasma protein that has a significant role in primary hemostasis. vWF is synthesized exclusively by endothelial cells and megakaryocytes as a pre-pro-vWF that consists of a 22 amino acid signal peptide, a 41 amino acid propeptide, and a mature subunit of 2,050 amino acids. After removal of the signal peptide, pro-vWF monomers dimerize in the endoplasmic reticulum through disulfide linkage at the C-terminal CK domain and multimerize the dimers in the Golgi complex via disulfide linkage of the N-terminal D3 domains. After synthesis, ultra-large von Willebrand factor (UL-VWF) is either constitutively secreted into the blood or stored in endothelial Weibel-Palade bodies and platelet α-granules. UL-vWF released at the apical surface can remain anchored on the surface of endothelial cells.^3^ Under the shear condition, such as at the AVS site, the UL-vWF opens up A1 and A2 domains. The opened A1 domains bind to the platelet glycoprotein Ib-IX-V complex and subsequently recruit platelets to this site. This accumulation of UL-vWF-platelet aggregation can be cleared by the vWF cleaving protease ADAMTS13 at the A2 domain. Digestion of UL-vWF by ADAMTS13 results in smaller, less active vWF multimers, which is also the underlying mechanism of acquired von Willebrand syndrome in patients with high-shear cardiovascular conditions, such as AVS. A deficiency of ADAMTS13 can cause thrombotic thrombocytopenic purpura.

Due to the anatomic proximity of AVD and AS and the current understanding of the role of UL-vWF–platelet interaction in AVD, Ozawa et al^4^ hypothesized that excess endothelial-associated vWF contributes to not only aortic valve thickening, but also the development of AVS through leaflet adhesion of platelets or platelet microvesicles and subsequent valve interstitial myofibroblastic and osteogenic transformation. They used a previously established mouse model that has both genetic defects of the low-density lipoprotein receptor (LDLR^−/−^) and ADAMTS13 (AD13^−/−^) and several unique techniques such as noninvasive in vivo imaging integrated with tissue histology to examine the effect of excess valve endothelial cell–released vWF on leaflet platelet adhesion, aortic valve morphology, valve interstitial cell transformation, valve hemodynamics, and load-dependent changes of the left ventricle.

In their elegant study in this issue of *JACC: Basic to Translational Science*, Ozawa et al^5^ first confirmed that their mouse model indeed reproduced the pathological phenotype of AVS. The blood pressure, aortic mechanical properties such as peak and median aortic gradients, aortic valve leaflet morphologies, and arterial afterload are all consistent with AVS. These findings further substantiate the speculation that AVS and AVD may share some pathophysiological similarities. Using imaging and histology studies, including transmission electron microscopy, they demonstrated that surface-anchored vWF and associated platelets or platelet microvesicles were increased at the apical surface of endothelial cells in the LDLR^−/−^/AD13^−/−^ mice. Interestingly, a histology study revealed the Mac-2–positive plaques were present in the aortic sinuses and extended into the base of the leaflets in the LDLR^−/−^/AD13^−/−^. However, the valve thickening was mainly caused by the increased matrix and cellularity. The matrix expansion was likely caused by collagen and active matrix-remodeling as assessed by trichome and MMP9 stains. The cellular proliferation was likely caused by myofibroblasts transformation as shown by α-smooth cell actin stains. In addition, valve calcification by calcium staining and osteogenic transformation by in situ hybridizations of osteopontin stain were only found in the LDLR^−/−^AD13^−/−^ mice. To explore the contribution of the adhered platelets, they evaluated the phosphorylated SMAD2 and β-catenin status of the TGFβ1 signaling pathway. The intensity and extent of expression for both phosphorylated SMAD2 and β-catenin were greater in LDLR^−/−^AD13^−/−^ mice than in other control groups, suggesting the potential mechanism of valve interstitial cell transformation and proliferation.

In summary, Ozawa et al^5^ tested the underlying hypothesis that excess endothelial-associated vWF and secondary platelet adhesion accelerate aortic valve thickening and calcification. The development of AVS likely involves a cascade of cellular and matrix events. The attached platelets may directly provide TGFβ1 and other factors to promote myofibroblast transformation and activation via the SMAD and Wnt/β-catenin pathways.

A couple of clinical implications are raised by this study. First, this study demonstrated that AVS and AVD likely share similar pathophysiology because both AVS and AVD can be reproduced using the same animal model. Second, vWF and platelets are likely the missing links to AVS disease initiation and progression. Lindner et al^5^ provided an appealing road map for the next phase of studies to see whether anti-vWF–platelet intervention could prevent AVS initiation or mitigate or even reverse the AVS progression, which ultimately would change the natural disease course of AVS. For example, the direct vWF A1–glycoprotein Ib platelet binding inhibitor, ARC1179,^6^ and other antiplatelet drugs could have an impact on the disease progression of AVS. The findings of Ozawa et al^5^ may benefit the understanding of the other cardiovascular conditions, such as the recently observed association between thrombotic thrombocytopenic purpura and cardiovascular diseases.

## Funding Support and Author Disclosures

The author has reported that he has no relationships relevant to the contents of this paper to disclose.
